# Zika Virus Disease Comparing Children and Adults in a Dengue-Endemic Setting

**DOI:** 10.4269/ajtmh.20-0795

**Published:** 2020-11-09

**Authors:** Jurai Wongsawat, Nutcharin Vivong, Patama Suttha, Sumonmal Utayamakul, Somtavil Aumpornareekul, Api Chewcharat, Kulkanya Chokephaibulkit

**Affiliations:** 1 Bamrasnaradura Infectious Diseases Institute, Department of Diseases Control, Ministry of Public Health, Nonthaburi, Thailand;; 2 Mount Auburn Hospital, Harvard Medical School, Cambridge, Massachusetts;; 3 Department of Pediatrics, Faculty of Medicine, Siriraj Hospital, Mahidol University, Bangkok, Thailand

## Abstract

Acute Zika virus (ZIKV) infection may mimic dengue virus (DENV) infection. We aimed to study the clinical difference of ZIKV disease among suspected non-severe DENV patients comparing children and adults. Patients with acute illness suspected of DENV disease plus no evidence of plasma leakage at the Bamrasnaradura Infectious Diseases Institute, Nonthaburi, Thailand, were enrolled from December 2016 to September 2018. Clinical data including DENV rapid diagnostic test (RDT) results were collected. Zika virus diagnosis was confirmed by real-time reverse transcription PCR on urine. Of 291 (180 pediatric and 111 adult) cases enrolled, 27 (10 pediatric and 17 adult) confirmed ZIKV cases were found. Rash was more frequent among pediatric ZIKV than pediatric non-ZIKV cases (100% versus 60%, *P =* 0*.*01). Rash, arthralgia, and conjunctivitis were more frequent among adult ZIKV than adult non-ZIKV cases (100% versus 29.8%, 64.7% versus 26.6%, 52.9% versus 9.7%, all *P <* 0*.*01, respectively). The median (interquartile range [IQR]) duration of rash was 4.5 (3.0, 7.25) days and 6.0 (4.5, 7.0) days in pediatric and adults ZIKV cases, respectively. Pediatric ZIKV cases had more fever (100% versus 58.5%, *P* = 0.03) but less arthralgia (20% versus 64.7%, *P =* 0*.*04) and less conjunctivitis (10% versus 52.9%, *P* = 0.04) than adult ZIKV cases. No ZIKV cases with DENV RDTs performed around day 3 of illness were positive for dengue nonstructural protein 1 (NS1) antigen. In dengue-endemic settings, rash and fever in children, and rash, arthralgia, and conjunctivitis in adults, particularly if rash persists for ≥ 3 days, plus negative dengue NS1 Ag during early febrile phase should prompt ZIKV diagnostic testing.

## INTRODUCTION

Mosquito-borne viral diseases have rapidly spread globally in the recent years. Dengue virus (DENV) and Zika virus (ZIKV) infections are mosquito-borne viral diseases that have become public health concerns worldwide because of their expanding geographic areas, the potential threat of severe clinical courses in DENV infections, as well as teratogenicity and spectrum of neuro-abnormalities in ZIKV infections.^1,2^

Both DENV and ZIKV are flaviviruses transmitted mainly by *Aedes* mosquito species, especially *Aedes aegypti*, and both share some similar clinical features. The complexity of serological diagnosis due to cross-reactivity of the antibodies among flaviviruses is disadvantageous, and specific molecular testing by real-time reverse transcription polymerase chain reaction (RT-PCR) is needed to confirm infection.^3^ Since 2015, ZIKV has attracted global concern because of a large epidemic in Latin America, associated with serious congenital neurological consequences and Guillain–Barre syndrome. It was declared as a public health emergency of international concern by the WHO on February 1, 2016^4^ and later was announced on November 18, 2016 to be incorporated into the longer term public response.^5^

According to the WHO, DENV is endemic in more than 100 countries, of which countries in the WHO regions of the Americas, Southeast Asia, and Western Pacific are the most seriously affected.^1^ As of July 2, 2019, the WHO reported ZIKV in 148 countries,^6^ some of which are also the most DENV-affected countries.

Dengue virus infection manifests with a diverse range of symptoms from mildly undifferentiated fever to severe diseases, although it may be asymptomatic in 70–80% of cases.^7^ According to the WHO, the clinical manifestations can be classified as dengue fever (DF) and severe dengue (previously known as dengue hemorrhagic fever), and suspected dengue is characterized by high fever accompanied by two of the following symptoms: headache, retrobulbar pain, nausea and vomiting, muscle and joint pain, and rash.^8^ Dengue fever has no apparent evidence of plasma leakage and is usually not a severe disease, whereas dengue hemorrhagic fever is characterized by vascular leakage and thrombocytopenia, which can lead to shock and bleeding.^9^

Zika virus disease may mimic non-severe forms of DENV disease. Overlapping clinical features between ZIKV and DENV cases make accurate diagnosis difficult, especially in an endemic arbovirus area.^10^ The clinical features of DF also vary according to the age of the patient.^8^ Furthermore, molecular testing is not routinely available in resource-limited settings where dengue is endemic because of its cost and sophisticated technique.^11^ Recently, commercial rapid diagnostic tests (RDTs) for DENV (DENV RDTs) including nonstructural protein 1 antigen (NS1 Ag) and anti-dengue IgM/IgG detection assays have become increasingly available for point-of-care testing. The degree of cross-reactivity between these tests with ZIKV is unclear.^12,13^ Differentiating ZIKV disease from other flavivirus diseases is desirable to provide appropriate management, apart from disease containment in the outbreak area, and comprehensive care for reproductive age women (15–49 years) is vital because it may help prevent serious morbidity associated with ZIKV infection including teratogenicity and neurological consequences, especially in the newborn. This study aimed to characterize ZIKV cases among suspected non-severe dengue cases, comparing children and adults and also the cross-reactivity of the commercial DENV RDTs with ZIKV cases. Combination of clinical characteristics plus DENV RDT approach could probably be used for ZIKV disease screening in resource-limited settings before molecular confirmation testing.

## METHODS

A prospective observational study was conducted from December 2016 to September 2018 at the Bamrasnaradura Infectious Diseases Institute, a tertiary care hospital and referral center for infectious diseases of the Department of Disease Control, Ministry of Public Health, Nonthaburi, Thailand.

All children (aged ≤ 15 years) and adult (aged > 15 years) patients presenting with acute illness suspected of DENV disease plus no evidence of plasma leakage (no rising of hematocrit ≥ 20% over baseline,^14^ assessed by pediatricians) were invited to join the study. The DENV RDTs were performed on an occasional basis based on clinician’s judgment in routine service. The enrollment criterion was meeting either clinical or DENV RDT (if performed) criteria given as follows: 1) Clinical criteria: having acute fever (≥ 37.8°C, within the previous 7 days) plus at least two of the following symptoms including headache, myalgia, rash (non-vesicular, non-pustular), or arthralgia; or 2) DENV RDTs criteria: having any positive result of DENV RDTs plus at least two of the following symptoms including acute fever (≥ 37.8°C, within the previous 7 days), headache, myalgia, rash (non-vesicular, non-pustular), or arthralgia. The commercial DENV RDTs available during the study period used an immunochromatography technique, which the manufacturers indicate sensitivity and specificity for dengue NS1 Ag ranging from 92.4% to 97.9% and from 98.4 to 99.0%, respectively, and sensitivity and specificity for anti-dengue IgM/IgG ranging from 85.1% to 98.8% and from 91.6 to 99.0%, respectively. Decisions for admission were also based on clinicians’s judgment. We did not enroll the patients with evidence of plasma leakage because this phenomenon is highly specific to the severe form of DENV infection and is rarely found in ZIKV infection.

After obtaining informed consent and assent in children older than 7 years, medical history and physical examinations were carried out. A standardized form was structured to collect clinical and laboratory data. A urine sample was then collected for specific ZIKV testing which, according to WHO recommendation,^3^ was collected within 7 days of symptoms onset. Urine was collected in a sterile and nuclease-free container and sent to the laboratory immediately, or otherwise stored at 4°C and within 24 hours shipped with icepack to the laboratory. One mililiter of urine was filtered and centrifuged at 25,000 revolutions/minute for 1 hour at 4°C. The supernatant was discarded, and the urine pellet was extracted using a QIAamp^®^ Viral RNA extraction kit (QIAGEN GmbH, Hilden, Germany) according to the manufacturer’s instruction. The extracted RNA was tested for specific ZIKV testing using a RealStar^®^ ZIKV RT-PCR Kit 1.0 (Altona, Hamburg, Germany). Positive and negative controls were included in every RT-PCR assay run. Testing was performed at the Bamrasnaradura Infectious Diseases Institute with the turnaround time for results of 2 working days. The enrolled patients were treated according to routine clinical practice in Thailand and were followed up until resolution of symptoms.

### Ethics approval.

This study was approved by the Ethics Committee of Bamrasnaradura Infectious Diseases Institute, Department of Disease Control, Ministry of Public Health, Thailand.

### Statistical analysis.

Descriptive data were analyzed by mean and SD for normally distributed data and median and interquartile range (IQR) for nonnormally distributed data. Clinical characteristics comparing 1) ZIKV and non-ZIKV cases in children and adults, and 2) pediatric and adults ZIKV cases were performed. Clinical comparisons between ZIKV and non-ZIKV cases among the cohort of negative dengue NS1 Ag (presumably excluded DENV infection) cases were also analyzed. The difference in proportions was compared using the Fisher exact or χ^2^ test. The differences in means and medians were compared using *t*-tests and the Mann–Whitney *U* tests, respectively. Univariable logistic regression was used to analyze the odds ratio [OR] for the associations of the clinical characteristics with the ZIKV infection. *P* < 0.05 was considered statistically significant. Analyses were undertaken using SPSS version 23 (IBM Corp., Armonk, NY).

## RESULTS

Two hundred ninety-one patients with clinically suspected non-severe DENV cases were enrolled, including 180 (62%) children (aged ≥ 15 years old) and 111 (38%) adults. One hundred forty (48%) cases were female, and 129 (44.3%) cases were treated as outpatients. Fifty-seven (19%) were women at reproductive age (15–49 years). Overall, the median (IQR) age was 12.0 (5.1, 29.0) years. The median (IQR) age was 6.4 (2.6, 11.0) years in children and 33.0 (25.3, 46.0) years in adults. The median (IQR) duration of illness at enrollment was 3.0 (2.0, 4.0) days and at urine sample collection for ZIKV testing was 5.0 (3.0, 7.0) days. Six pregnant women were enrolled in the study. Of the 263 (90.4%) cases who had available DENV RDTs results comprising 51 (19.4%) dengue NS1 Ag, 32 (12.2%) anti-dengue IgM/IgG, and 180 (68.4%) both dengue NS1 Ag and anti-dengue IgM/IgG, we found that 96 (41.5%) of 231, and 65 (30.6%)/91 (42.9%) of 212 were positive for dengue NS1 Ag and anti-dengue IgM/IgG, respectively. The median (IQR) duration of illness at DENV RDTs performed was 3.0 (2.0, 4.0) days.

Of the 291 enrolled cases, ZIKV RNA was positive in urine samples of 27 (9.3%) patients, of which 10 (37%) were children and 17 (63%) were adults; none were pregnant women. Most ZIKV cases occurred between August 2017 and January 2018. Table 1 shows the overall characteristics and comparison of ZIKV and non-ZIKV cases. The most common symptoms in ZIKV cases were rash (100%), followed by fever (74.1%), arthralgia (48.1%), headache (44.4%), myalgia (44.4%), and non-purulent conjunctivitis (37.0%). The symptoms that were significantly more frequent in ZIKV cases than non-ZIKV cases were rash (100.0% versus 49.2%; *P* < 0.01), arthralgia (48.1% versus 16.1%; *P* < 0.01), and conjunctivitis (37.0% versus 6.8%; *P* < 0.01), although fever was less frequent (74.1% versus 98.1%; *P* < 0.01). Of the 13 and 10 ZIKV cases with arthralgia and conjunctivitis, the median (IQR) total duration of symptoms was four (1.5, 6.5) days and 2.5 (1.75, 4.0) days, respectively. Complete blood count performed at the median (IQR) 3.0 (2.0, 4.0) days of illness revealed no significant differences between ZIKV and non-ZIKV cases. None of the ZIKV cases had positive dengue NS1 Ag or anti-dengue IgM results, whereas 45.3% and 32.8% of the non-ZIKV cases had positive results by each test, respectively (both *P* < 0.01, Fisher’s exact test). No significant difference for the percentage of positive anti-dengue IgG between ZIKV (35.7%) and non-ZIKV (43.4%) cases (*P* = 0.78).

**Table 1:** Clinical characteristics comparing ZIKV and non-ZIKV cases

	ZIKV (*N* = 27)	Non-ZIKV (*N* = 264)	*P*-value	OR (95% CI)
Female	16 (59.3)*	124 (47.0)	0.23	1.64 (0.73–3.67)
Fever	20 (74.1)	259 (98.1)	**< 0.01**	0.06 (0.02–0.19)
Rash	27 (100)	130 (49.2)	**< 0.01**	NA
Headache	12 (44.4)	146 (64.0); 36†	0.06	0.45 (0.20–1.01)
Myalgia	12 (44.4)	123 (53.7); 36	0.42	0.69 (0.31–1.54)
Arthralgia	13 (48.1)	37 (16.1); 36	**< 0.01**	4.84 (2.11–11.14)
Conjunctivitis	10 (37.0)	18 (6.8)	**< 0.01**	8.01 (3.20–20.01)
Rhinorrhea/sore throat	6 (22.2)	61 (23.1)	1.00	0.95 (0.37–2.46)
Cough	2 (7.4)	75 (28.4)	**0.02**	0.20 (0.05–0.87)
Diarrhea	1 (3.7)	35 (13.8); 10‡	0.22	0.24 (0.03–1.83)
Positive RDT dengue nonstructural protein 1 Ag	0 (0.0); 8§	96 (45.3); 52	**< 0.01**	NA
Positive RDT dengue IgM	0 (0); 13	65 (32.8); 66	**< 0.01**	NA
Positive RDT dengue IgG	5 (35.7); 13	86 (43.4); 66	0.78	0.72 (0.23–2.24)
Duration of illness at enrollment, median (IQR), days	3.0 (1.0, 4.0)	3.0 (2.0, 4.0)	0.16	NA
Duration of fever, median (IQR), days	3.0 (2.0, 4.75); 20^‖^	5.0 (4.0, 6.0); 259	**0.01**	NA
Duration of rash, median (IQR), days	5.0 (4.0, 7.0); 27	3.5 (2.0, 5.0); 130	**< 0.01**	NA
Duration of headache, median (IQR), days	1.0 (1.0, 1.75); 12	1.0 (1.0, 3.0); 146	0.29	NA
Duration of myalgia, median (IQR), days	1.0 (1.0, 2.75); 12	2.0 (1.0, 3.0); 123	0.48	NA
Duration of arthralgia, median (IQR), days	4.0 (1.5, 6.5); 13	1.0 (0.5, 3.0); 37	**0.01**	NA
Duration of conjunctivitis, median (IQR), days	2.5 (1.75, 4.0); 10	2.0 (1.0, 4.0); 18	0.52	NA
Age, mean (SD), years	27.4 (16.5)	17.1 (17.0)	**< 0.01**	NA
Body weight, mean (SD), kg	56.4 (19.0)	40.9 (29.2)	**< 0.01**	NA
Peak temperature, mean (SD), °C	37.9 (0.68)	38.9 (0.89)	**< 0.01**	NA
Hematocrit, mean (SD), %	36.5 (11.1)	39.3 (19.4)	0.49	NA
White blood cell, mean (SD), 10^9^/L	7.2 (0.8)	6.6 (3.9)	0.6	NA
Platelets, mean (SD), 10^9^/L	235 (78.6)	212 (88.2)	0.2	NA

IQR = interquartile range; NA = not applicable; OR = odds ratio; RDT = rapid diagnostic test; ZIKV = Zika virus. Bold values indicate significant difference (*P* < 0.05).

* No. (%).

† No. (%); no. of cases that could not be evaluated because of young age (< 2 years).

‡ No. (%) yes; no. of missing.

§ No. (%) yes; no. of RDT not performed.

‖ Median (IQR); no. of cases.

Among the 180 child cases (10 ZIKV and 170 non-ZIKV cases), rash was the only symptom found significantly more commonly in ZIKV cases than non-ZIKV cases (100.0% versus 60.0%; *P* = 0.01) (Table 2). The positive predictive value (PPV) of rash for ZIKV infection was 8.9%. Of the eight ZIKV cases with maculopapular rash, four had pruritus. Scarlatiniform rash was present in the other two ZIKV cases. Peak temperature was significantly lower (38.1°C versus 39.0°C; *P* < 0.01) with a shorter duration of fever (3 days versus 5 days; *P* < 0.01) in ZIKV than non-ZIKV cases, respectively. No combined clinical characteristics were significantly predictive of ZIKV disease in this age-group.

**Table 2:** Clinical characteristics comparing ZIKV and non-ZIKV cases in children and adults

	Children (*N* = 180)				Adults (*N* = 111)				Compare ZIKV cases (10 children, 17 adults)	
	ZIKV (*N* = 10)	Non-ZIKV (*N* = 170)	*P*-value	OR (95% CI)	ZIKV (*N* = 17)	Non-ZIKV (*N* = 94)	*P*-value	OR (95% CI)	*P*-value	OR (95% CI)
Female	6 (60.0)*	65 (38.2)	0.19	0.41 (0.11–1.52)	10 (58.8)*	59 (62.8)	0.79	1.18 (0.41–3.38)	1.0	0.95 (0.19–4.67)
Fever	10 (100)	167 (98.2)	1.00	NA	10 (58.8)	92 (97.9)	**< 0.01**	0.03 (0.01–0.17)	**0.03**	NA
Rash	10 (100)	102 (60.0)	**0.01**	NA	17 (100)	28 (29.8)	**< 0.01**	NA	NA	NA
Headache	5 (50.0)	68 (50.7); 36†	1.00	0.97 (0.27–3.51)	7 (41.2)	78 (83.0)	**< 0.01**	0.14 (0.05–0.43)	0.71	1.43 (0.29–6.87)
Myalgia	3 (30.0)	41 (30.4); 36	1.00	0.98 (0.24–3.99)	9 (52.9)	82 (87.2)	**< 0.01**	0.17 (0.05–0.51)	0.42	0.38 (0.07–1.99)
Arthralgia	2 (20.0)	12 (8.8); 36	0.24	2.60 (0.49–13.68)	11 (64.7)	25 (26.6)	**< 0.01**	4.99 (1.67–14.91)	**0.04**	0.14 (0.02–0.86)
Conjunctivitis	1 (10.0)	9 (5.3)	0.44	1.98 (0.23–17.45)	9 (52.9)	9 (9.7)	**< 0.01**	10.5 (3.24–33.99)	**0.04**	0.09 (0.01–0.96)
Rhinorrhea/sore throat	3 (30.0)	44 (25.9)	0.72	1.23 (0.30–4.95)	3 (17.6)	17 (18.1)	1.00	0.97 (0.25–3.75)	0.64	2.0 (0.32–12.59)
Cough	2 (20.0)	54 (31.8)	0.73	0.54 (0.11–2.61)	0 (0)	21 (22.3)	**0.04**	NA	0.13	NA
Diarrhea	0 (0)	24 (15.0); 10‡	0.36	NA	1 (5.9)	11 (11.7)	0.69	0.47 (0.06–3.91)	1.00	NA
Positive RDT dengue nonstructural protein 1 Ag	0 (0); 4§	59 (45.4); 40	**0.04**	NA	0 (0); 4§	37 (45.1); 12	**< 0.01**	NA	NA	NA
Positive RDT dengue IgM	0 (0); 7	41 (36.0); 56	0.55	NA	0 (0); 6	24 (28.6); 10	0.06	NA	NA	NA
Positive RDT dengue IgG	1 (33.3); 7	33 (28.9); 56	1.00	1.23 (0.11–14.00)	4 (36.4); 6	53 (63.1); 10	0.11	0.33 (0.09–1.23)	1.00	0.87 (0.06–12.97)
Duration of illness at enrollment, median (IQR), days	3.0 (1.75, 4.0)	3.0 (2.0, 4.0)	0.43	NA	2.0 (1.0, 3.5)	3.0 (2.0, 4.0)	0.27	NA	0.72	NA
Duration of fever, median (IQR), days	3.0 (2.0, 4.25); 10^‖^	5.0 (4.0, 6.0); 167	**< 0.01**	NA	3.0 (2.0, 5.24); 10	5.0 (3.0, 6.0); 92	0.08	NA	0.64	NA
Duration of rash, median (IQR), days	4.5 (3.0, 7.25); 10	4.0 (3.0, 5.0); 102	0.17	NA	6.0 (4.5, 7.0); 17	3.0 (2.0, 4.75); 28	**< 0.01**	NA	0.15	NA
Duration of headache, median (IQR), days	1.0 (1.0,1.0); 5	2.0 (1.0, 3.0); 68	0.07	NA	1.0 (1.0, 7.0); 7	1.0 (1.0, 3.0); 78	0.85	NA	0.11	NA
Duration of myalgia, median (IQR), days	1.0 (1.0, 1.0); 3	2.0 (1.0, 4.0); 41	0.19	NA	1.0 (1.0, 4.0); 9	1.5 (1.0, 3.0); 82	0.96	NA	0.18	NA
Duration of arthralgia, median (IQR), days	2.0 (2.0, 2.0); 2	1.0 (0.5, 2.75); 12	0.70	NA	4.0 (2.0, 7.0); 11	1.0 (1.0, 3.0); 25	**0.02**	NA	0.37	NA
Duration of conjunctivitis, median (IQR), days	3.0 (3.0, 3.0); 1	2.0 (2.0, 3.0); 9	0.47	NA	2.0 (1.5, 4.0); 9	2.0 (1.0, 4.5); 9	0.62	NA	0.72	NA
Age, mean (SD), years	10.0 (3.19)	6.58 (4.58)	**0.02**	NA	37.7 (11.6)	36.0 (14.6)	0.65	NA	NA	NA
Body weight, mean (SD), kg	39.6 (14.6)	28.5 (27.8)	0.21	NA	66.3 (13.4)	63.6 (14.5)	0.47	NA	NA	NA
Peak temperature, mean (SD), °C	38.1 (0.60)	39.0 (0.89)	**< 0.01**	NA	37.6 (0.67)	38.6 (0.84)	< 0.01	NA	0.06	NA
Hematocrit, mean (SD), %	34.6 (12.3)	39.3 (23.2)	0.53	NA	37.9 (10.6)	39.6 (5.58)	0.34	NA	0.49	NA
White blood cell, mean (SD), 10^9^/L	5.2 (0.9)	6.9 (3.9)	0.18	NA	8.5 (2.7)	6.2 (3.6)	0.11	NA	0.36	NA
Platelets, mean (SD), 10^9^/L	244 (73.5)	223 (90.6)	0.48	NA	228 (83.9)	187 (76.6)	0.06	NA	0.64	NA

IQR = interquartile range; NA = not applicable; OR = odds ratio; RDT = rapid diagnostic test; ZIKV = Zika virus.

* No. (%).

† No. (%); no. of cases that could not be evaluated because of young age (less than 2 years).

‡ No. (%) yes; no. of missing.

§ No. (%) yes; no. of RDT not performed.

‖ Median (IQR); no. of cases.

Among 111 adult cases (17 ZIKV and 94 non-ZIKV), rash (100.0% versus 29.8%; *P* < 0.01), arthralgia (64.7% versus 26.6%; *P* < 0.01), and conjunctivitis (52.9% versus 9.7%; *P* < 0.01) were significantly found more common in ZIKV cases than non-ZIKV cases (Table 2). The PPV of rash for ZIKV infection was 37.7%. All rashes were maculopapular, and 70% of them were accompanied by pruritus. The duration of rash was significantly longer among ZIKV cases than non-ZIKV cases (median [IQR] 6 days [4.5, 7.0] versus 3 days [2.0, 4.75]; *P* < 0.01). Fever was significantly less common (58.8% versus 97.9%; *P* < 0.01), peak temperature was lower (37.6°C versus 38.6°C; *P* < 0.01), and duration of fever was shorter (median [IQR] 3 days [2.0, 5.25] versus 5 days [3.0, 6.0]; *P* = 0.08) among ZIKV cases than non-ZIKV cases. Headache (41.2% versus 83.0%; *P* < 0.01), myalgia (52.9% versus 87.2%; *P* < 0.01), and cough (0.0% versus 22.3%; *P* = 0.04) were significantly less common in ZIKV cases. Combined clinical characteristics including rash plus fever plus conjunctivitis plus arthralgia (OR [95% CI], 9.33 [1.87–46.49]; *P* = 0.01) were predictive for ZIKV disease in adult, giving the PPV of 57.1%.

Comparison of the clinical characteristics of the 10 pediatric and 17 adult ZIKV cases is shown in Table 2. All cases presented with rash, of which the most common (93%) was maculopapular, 17 (63%) were accompanied by various degrees of pruritus. Neither the frequency of itching (50% versus 70%; *P* = 0.42) nor the duration of rash (median 4.5 days versus 6.0 days; *P* = 0.15) was significantly different between the child and adult ZIKV cases. Pediatric ZIKV cases had fever more frequently (100% versus 58.8%; *P* = 0.03) with a higher peak temperature than adult ZIKV cases (38.1°C versus 37.6°C; *P* = 0.06). Adult ZIKV cases significantly had arthralgia (64.7% versus 20%; *P* = 0.04) and conjunctivitis (52.9% versus 10%; *P* = 0.04) more frequently than pediatric ZIKV cases. Among the 13 cases (two children and 11 adults) with arthralgia, the median (IQR) duration was 4.0 days (1.5, 6.5), and the most commonly affected joints were ankle and knee and usually were multi-joint involvement. Two adult ZIKV cases also developed mildly swollen joints.

Sub-analysis of clinical comparisons between ZIKV and non-ZIKV cases for 135 negative RDT dengue NS1 Ag (presumably excluded DENV infection) cases was performed (Table 3). Among 77 child cases (six ZIKV and 71 non-ZIKV cases), no significant differences for the frequency of symptoms were found, although ZIKV cases were older (9.3 years versus 5.2 years; *P* = 0.03) and revealed lower fever (38.0°C versus 38.9°C; *P* = 0.03) than non-ZIKV cases, The PPV of rash for ZIKV infection was 11.3%. We also observed shorter duration of fever (median [IQR] 2.5 days [2.0, 4.0] versus 4.0 days [4.0, 6.0]; *P* = 0.02) but longer duration of rash (median [IQR] 3.5 days [3.0, 8.0] versus 2.0 days [0.5, 4.0]; *P* = 0.04) in ZIKV cases than non-ZIKV cases. Among 58 adult cases (13 ZIKV and 45 non-ZIKV), rash (100.0% versus 28.9%; < 0.01), arthralgia (69.2% versus 28.9%; *P* = 02), and conjunctivitis (53.8% versus 8.9%; < 0.01) were significantly found more common, whereas fever was significantly less common (53.8% versus 95.6%; *P* < 0.01) and revealed lower degree (37.5°C versus 38.5°C; *P* < 0.01) in ZIKV cases than non-ZIKV cases. The PPV of rash for ZIKV infection was 50%. The median [IQR] duration of rash and arthralgia was also longer (6.0 days [4.5, 7.0] versus 3.0 days [2.0, 5.0], and 4.0 days [1.5, 6.5] versus 1.0 days [0.5, 4.0]; both *P* = < 0.01, respectively) in ZIKV cases than non-ZIKV cases. Combined clinical characteristics including rash plus fever plus conjunctivitis plus arthralgia (OR [95% CI], 13.20 [1.24–140.51]; *P* = 0.03) were predictive for ZIKV disease in adult, giving the PPV of 75.0%.

**Table 3:** Selected clinical comparisons between ZIKV and non-ZIKV cases, among 135 negative rapid diagnostic test dengue nonstructural protein 1 Ag cases

	Children (*N* = 77)				Adults (*N* = 58)			
	ZIKV (*N* = 6)	Non-ZIKV (*N* = 71)	*P*-value	OR (95% CI)	ZIKV (*N* = 13)	Non-ZIKV (*N* = 45)	*P*-value	OR (95% CI)
Age, mean (SD), years	9.3 (3.8)	5.2 (4.4)	**0.03**	NA	36.2 (10.3)	38.5 (15.6)	0.62	NA
Peak temperature, mean (SD), °C	38.0 (0.75)	38.9 (0.9)	**0.03**	NA	37.5 (0.6)	38.5 (0.8)	**< 0.01**	NA
Fever	6 (100)*	71 (100)	NA	NA	7 (53.8)	43 (95.6)	**< 0.01**	0.05 (0.01–0.32)
Rash	6 (100)	47 (66.2)	0.17	NA	13 (100)	13 (28.9)	**< 0.01**	NA
Headache	2 (33.3)	25 (51.0); 22†	0.67	0.48 (0.08–2.87)	7 (53.8)	38 (84.4)	0.05	0.21 (0.05–0.83)
Myalgia	1 (16.7)	16 (32.0); 21	0.65	0.43 (0.05–3.94)	8 (61.5)	38 (84.4)	0.12	0.29 (0.07–1.17)
Arthralgia	0 (0.0)	4 (7.8); 20	1.00	NA	9 (69.2)	13 (28.9)	**0.02**	5.53 (1.45–21.21)
Conjunctivitis	1 (16.7)	2 (2.8)	0.22	6.9 (0.53–89.83)	7 (53.8)	4 (8.9)	**< 0.01**	11.96 (2.67–53.47)
Rhinorrhea/sore throat	1 (16.7)	19 (26.8)	1.00	0.54 (0.06–4.99)	3 (23.1)	11 (24.2)	1.00	0.93 (0.22–3.98)
Cough	1 (16.7)	23 (32.4)	0.66	0.42 (0.05–3.78)	0 (0)	12 (26.7)	0.05	NA
Diarrhea	0 (0)	8 (12.7); 8‡	1.00	NA	1 (7.7)	5 (11.1)	1.00	0.67 (0.07–6.27)

NA = not applicable; OR = odds ratio; ZIKV = Zika virus.

* No. (%).

† No. (%); no. of cases that could not be evaluated because of young age (less than 2 years).

‡ No. (%) yes; no. of missing.

## DISCUSSION

Zika virus infection can cause a clinical illness that mimics mild DENV infection. The eligibility criteria in our study reflect real clinical practices in resource-limited settings where molecular testing is not routinely performed to confirm DENV infection, and most suspected DENV cases are diagnosed by clinically compatible symptoms plus positive DENV RDTs testing. All ZIKV cases in our study presented with rash, most commonly maculopapular rash. Compared with non-ZIKV cases, ZIKV cases were more likely to have rash, arthralgia, non-purulent conjunctivitis, and milder fever and were less likely to have cough. These findings were compatible with previous studies.^15–17^ The long median duration of rash (5 days) and arthralgia (4 days) among ZIKV cases in our study was also similar to a previous report.^15^ Around 50–70% of our ZIKV cases had pruritus, which suggests that pruritus should be added to the ZIKV case definitions.^17,18^

Previous studies found that rash and fever were the two most common symptoms in pediatric ZIKV cases.^19,20^ However, rash was the only symptom that was significantly more common in children with ZIKV than those without ZIKV in our study. All pediatric ZIKV cases in our study had lower peak temperature than pediatric non-ZIKV cases. We found a lower proportion of conjunctivitis than those reported in another study (10% versus 58%).^21^ We also observed significantly older age among pediatric ZIKV cases than pediatric non-ZIKV cases, which may reflect the higher chance of exposure to ZIKV-infected mosquitoes among the older children possibly through increased frequency of outdoor activities.

Comparing ZIKV cases with non-ZIKV cases in adults, rash, arthralgia, and conjunctivitis were significantly more common, whereas fever, headache, myalgia, and cough were less common. These findings were also compatible with previous studies, of which majority were adults.^15–17^ Conjunctivitis was found in about half of the adult ZIKV cases in our study similar to some previous studies, and this suggests that conjunctivitis should be included in the definition of ZIKV disease in adults.^17,22^

Taken into account for sub-analysis among the cases with negative dengue NS1 Ag which presumably were less likely to be DENV infection, it revealed that in the pediatric age-group, we cannot differentiate ZIKV infection from other viral infections because no significant differences for the frequency of symptoms were seen, although less intensity of fever and longer duration of rash may support ZIKV infection in this age-group. For the adult age-group, rash, arthralgia, and conjunctivitis were still significantly more common in ZIKV than non-ZIKV cases.

Our findings support the WHO’s surveillance criteria for suspected ZIKV infection,^23^ which indicate a person presenting with rash and/or fever and at least one of the following symptoms including arthralgia, arthritis, or conjunctivitis, although these criteria seem to be more predictive for ZIKV disease in adults than children. For children, because of the similarity of symptoms between ZIKV infection and other viral infections, apart from negative RDT dengue NS1 Ag, the surveillance criteria for suspected ZIKV infection in this age-group may need epidemiologic link with the index cases in the outbreak areas.

Our findings also support the Thailand’s surveillance criteria for suspected ZIKV disease, which indicate a person presenting with rash and at least one of the following including fever, arthralgia, headache, and conjunctivitis; and if negative RDT dengue NS1 Ag during the first 3 days of illness is documented, the case would be classified as probable ZIKV infection accordingly.

Our study adds to the limited knowledge about the difference between ZIKV disease in children and adults. As with other previous studies, we found that ZIKV disease was mild in both children and adults.^20,24^ However, we found that pediatric ZIKV cases tended to more frequently manifest fever and higher fever but less frequently manifest arthralgia and conjunctivitis than adult ZIKV cases. A previous study from the United States also reported that arthralgia was more common in adults.^20^ The reasons for the discrepancy of ZIKV characteristics between children and adults are unclear but could be because of the different host responses among different age-groups.

In our study, the routine complete blood count on day 3 of illness did not differentiate between ZIKV and non-ZIKV cases among suspected non-severe DENV infection cases. However, our eligibility criteria excluded suspected DENV cases with severe symptoms, which are likely to have more profound leukopenia and thrombocytopenia.

Dengue RDTs are now commonly used in clinical settings to help diagnose DENV infection, although they do not confirm infection. A false-positive dengue NS1 Ag test in a traveler with ZIKV infection was previously reported.^25^ In our study, at the median duration of 3 days of illness, we found no positive dengue NS1 Ag or anti-dengue IgM in any patients with ZIKV disease in both children and adults, although 36% had positive anti-dengue IgG. Our findings support previous studies that found a high specificity of the RDT dengue NS1 Ag with limited to no cross-reactivity with ZIKV.^26,27^

There were some limitations in this study. There may have been some patients with ZIKV and DENV coinfections or even chikungunya infections that were not diagnosed because only RT-PCR for ZIKV was performed. We did not enroll suspected DENV cases with evidence of plasma leakage or severe symptoms because these patients would be less likely to have ZIKV infections. Therefore, we may have missed unusually more severe ZIKV cases, or cases with ZIKV and DENV coinfections. Because the collection of blood for DENV RDTs and urine for specific ZIKV testing was at separate time points, and not all cases had DENV RDTs performed, further study may be needed to better understand the cross-reactivity of the commercial DENV RDTs with ZIKV cases.

In conclusion, we found that in a dengue-endemic setting, among suspected non-severe DENV cases, rash and low-grade fever in children and rash plus arthralgia and conjunctivitis in adults, particularly if the rash is maculopapular and persists for more than 3 days, plus negative RDT dengue NS1 Ag test during the acute phase of illness may lead to suspicion of ZIKV infection and laboratory confirmation would then be needed.
